# Resolutions of Respect to Dr. Winder

**Published:** 1894-09

**Authors:** 


### RESOLUTIONS OF RESPECT TO DR. WINDER.

At the regular meeting of the New Jersey State Dental Association, held at Asbury Park, July 19, 1894, the following resolutions were passed:

Whereas, In the death of our esteemed honorary member, Richard B. Winder, M.D., D.D.S., Dean of the Baltimore College of Dental Surgery, the dental profession and this Association have lost one of its worthy and honored members. He was foremost with those who labored to advance the dental profession by filling its ranks with honorable dentists. He was a devoted professional brother, kind and charitable to an eminent degree, and filled with true Southern hospitality wherever met. Therefore be it

*Resolved*, That we bow to the inevitable, yet desire to emphasize our grief at his death, and to bear testimony to his many abilities and self-sacrificing spirit in his intercourse with his fellow-men.

*Resolved*, That a copy of these resolutions be spread on the minutes and the same be sent to the dental journals for publication.

F. C. Barlow.
S. C. G. Watkins.
R. M. Sanger.

